# Hepatoprotective Effect of *Millettia dielsiana*: In Vitro and In Silico Study

**DOI:** 10.3390/molecules27248978

**Published:** 2022-12-16

**Authors:** Vu Thi Thu Le, Dao Viet Hung, Bui Minh Quy, Pham Thi Hong Minh, Do Tien Lam

**Affiliations:** 1 Thai Nguyen University of Agriculture and Forestry, Thai Nguyen University (TNU), Quyet Thang, Thai Nguyen 24119, Vietnam; 2 Thai Nguyen University of Sciences, Thai Nguyen University (TNU), Tan Thinh, Thai Nguyen 24119, Vietnam; 3 Institute of Natural Products Chemistry, Vietnam Academy of Science and Technology (VAST), 18 Hoang Quoc Viet, Cau Giay, Hanoi 10072, Vietnam; 4 Graduate University of Science and Technology, Vietnam Academy of Science and Technology (VAST), 18 Hoang Quoc Viet, Cau Giay, Hanoi 10072, Vietnam

**Keywords:** *Millettia dielsiana*, *Hepatocellular carcinoma*, antioxidant activity

## Abstract

In silico docking studies of 50 selected compounds from *Millettia dielsiana* Harms ex Diels (family Leguminosae) were docked into the binding pocket of the PI3K/mTOR protein. In there, compounds *trans*−3−*O-p*-hydroxycinnamoyl ursolic acid (**1**) and 5,7,4′−trihydroxyisoflavone 7−*O*−*β*−D−apiofuranosyl−(1→6)−*β*−D−glucopyranoside (**2**) are predicted to be very promising inhibitors against PI3K/mTOR. They direct their cytotoxic activity against *Hepatocellular carcinoma* with binding affinity (BA) values, the pulling work spent to the co-crystallized ligand from the binding site of PI3K/mTOR (W and F_max_), and the non-equilibrium binding free energy (∆G_neq_^Jar^) as BA values = −9.237 and −9.083 kcal/mol, W = 83.5 ± 10.6 kcal/mol with F_max_ = 336.2 ± 45.3 pN and 126.6 ± 21.7 kcal/mol with F_max_ = 430.3 ± 84.0 pN, and ∆G_neq_^Jar^ = −69.86074 and −101.2317 kcal/mol, respectively. In molecular dynamic simulation, the RMSD value of the PI3K/mTOR complex with compounds (**1** and **2**) was in the range of 0.3 nm to the end of the simulation. Therefore, the compounds (**1** and **2**) are predicted to be very promising inhibitors against PI3K/mTOR. The crude extract, ethyl acetate fraction and compounds (**1** and **2**) from *Millettia dielsiana* exhibited moderate to potent in vitro cytotoxicity on *Hepatocellular carcinoma* cell line with IC_50_ values of 81.2 µg/mL, 60.4 µg/mL, 23.1 μM, and 16.3 μM, respectively, and showed relatively potent to potent in vitro antioxidant activity on mouse hepatocytes with ED_50_ values of 24.4 µg/mL, 19.3 µg/mL, 30.7 μM, and 20.5 μM, respectively. In conclusion, *Millettia dielsiana* and compounds (**1** and **2**) are predicted to have very promising cytotoxic activity against *Hepatocellular carcinoma* and have a hepatoprotective effect.

## 1. Introduction

*Hepatocellular carcinoma* (HCC) is a fairly common malignancy of the digestive system, causing the most damage of all types of liver cancer. This cancer is very common, accounting for 80–90% of liver cancer cases because it arises in the background of a liver that has been damaged by viral hepatitis, alcohol, fat, type 2 diabetes, and iron pigment, especially when the liver has cirrhosis [1,2]. Worldwide, this is the 5th most common cancer and the 2nd leading cause of cancer-related death [1,3]. Currently, *Hepatocellular carcinoma* accounts for 75–85% of cancer-related deaths, of which the highest concentration is in men and the elderly. This cancer is most common in the countries of East Asia, Southeast Asia, and Northwest Africa [4]. There are many risk factors associated with *Hepatocellular carcinoma,* such as hepatitis (B and C), alcohol, tobacco, aflatoxin, aristolochic acid, etc., but hepatitis B and alcohol are the two main causes of the disease [5]. In the treatment of cancer in general and liver cancer in particular, drugs are used and appreciated because they do not damage normal cells and cause little harm to the body. However, the target drugs when treating liver cancer all bring unwanted side effects [6,7]. Numerous studies have proved the therapeutic potential of several phytochemicals against a variety of diseases, including cancer, because active plant-derived substances suppress carcinogenesis by protecting cells and regulating cell death. Therefore, the anti-cancer drugs derived from herbs are more and more interesting [7].

*Millettia dielsiana* Harms ex Diels is a species of woody vine belonging to the plant Leguminosae family and widely used in traditional medicine for a variety of treatments or cures for anemia, arthritis, as a sedative or to induce sleep, etc. [8]. It grows in evergreen closed forests and sometimes in semi-arid or semi-deciduous forests, and is distributed mainly in Laos, China, and Vietnam [9]. According to many previous studies, flavonoids and terpenoids are the chemical constituents of this plant with antitumor activity and hepatoprotective effects; there are also triterpenoids, alkaloids, steroids, etc. [10,11,12,13]. In addition to the remarkable activity above, the excellent safety profile also makes flavonoids the most valuable active ingredient for cancer prevention compared to the use of drugs and chemotherapy. Flavonoid and triterpenoid compounds can interfere with cancer cell proliferation, metastasis, and cell cycle arrest, and can promote cell differentiation, angiogenesis, and malignant cell death. The use of plant-based ingredients helps to prevent side effects and also create a better therapeutic effect [14]. 

The mechanistic target of rapamycin (mTOR) is a protein kinase that plays a central role in the regulation of cell metabolism, growth, and proliferation belonging to the phosphoinositide3-kinase (PI3K) family [15,16]. Many studies have also shown that the mTOR signaling pathway can promote tumor emergence and progression by regulating tumor cell death and apoptosis [17]. mTOR is an important pathway in *Hepatocellular carcinoma* that is linked to less-differentiated tumors, bad prognosis, and earlier recurrence independently of the underlying etiology of liver cancer [18]. The action of mTOR takes place when nutrients are sufficiently replenished to promote assimilation, energy storage, and efficient use. In addition, a series of novel inhibitors have shown high anti-tumor activity in clinical studies, and the use of anti-tumor drugs inhibiting mTOR in combination with other anti-tumor drugs has significant effects [10]. However, mTOR inhibitors still have side effects and poor pharmacokinetic characteristics, so clinical management is still needed in changing the dosage to safely use these drugs [17].

## 2. Results and Discussion

### 2.1. In Silico Docking of Isolated Compounds from Millettia dielsiana against PI3K/mTOR

**Figure 1:**
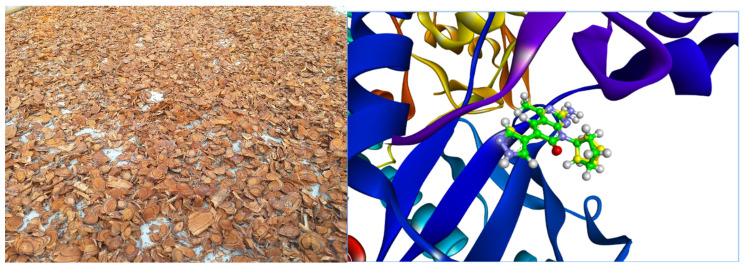
The stems of *Millettia dielsiana* and docking of the co-crystallized ligand to the PI3Kα/mTOR structure 4FA6. The initial ligand is in green and the docked ligand is in yellow.

The discovery and screening of agents derived from natural medicinal plants against cancer plays an important role in current drug development. The study of molecular docking was chosen as the first step in screening biologically active compounds. To investigate the potential efficacy of compounds isolated from *Millettia dielsiana*, molecular docking was performed against PI3K and mTOR proteins. Re-docking research is done to check the reliability of a docking program. The highest-ranked poses predicted with AutoDock Vina v1.2.3 are similar to the crystal-bonded poses of the reference ligand, according to the re-docking results (Figure 1). The root-mean-square deviation (RMSD) value of AutoDock Vina’s reconstructed crystal pose is 0.3478 Å, proving that our docking protocol is reliable. Therefore, AutoDock Vina was used to predict the binding affinities and molecular properties of the studied compounds complexed with PI3K/mTOR.

**Figure 2:**
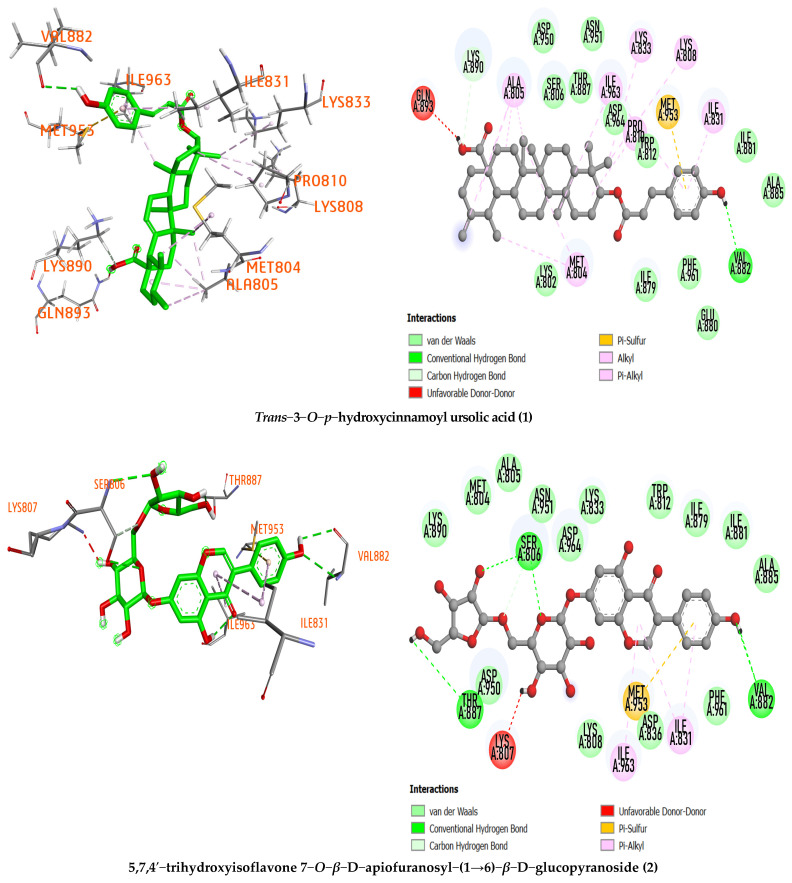

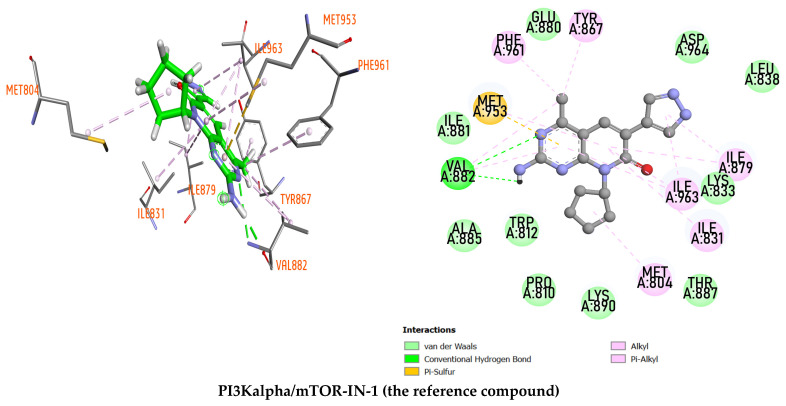
The 3D and 2D representations of the interactions of the studied compound inside the active sites of the PI3Kα/mTOR target protein.

A total of 50 compounds isolated from *Millettia dielsiana* were docked into the binding pocket of the PI3K/mTOR protein. Table 1 shows the ligand–receptor interactions within the target binding site and the overall interactions of the promising binding compounds versus the co-crystallized ligand. From this comparison, it was illustrated that the compound *trans*−3−*O*−*p*−hydroxycinnamoyl ursolic acid (**1**) had a co-crystallized ligand-like interaction, with Val 882 being the important amino acid in the 4FA6 binding pocket (Figure 2). This suggests similar inhibitory effects on PI3K and mTOR as co-crystallization ligands.

Furthermore, the compound *trans*−3−*O*−*p*−hydroxycinnamoyl ursolic acid (**1**) was considered the best-docked compound compared with the reference compound based on its low binding affinity (−9.237 kcal/mol), hydrogen bond formation with important amino acid Val882 (with a distance of 2.45 Å) and hydrophobic interactions with various amino acid residues with the target protein binding pocket, including MET804, LYS833, LYS808, ILE831, MET953, ILE963, PRO810, LYS890, and ALA805. On the other hand, other selected compounds have no higher binding affinity than the reference compound. Therefore, compound **1** deserves further testing to confirm its mode of action (Table 1).

For compound 5,7,4′−trihydroxyisoflavone 7−*O*−*β*−D−apiofuranosyl−(1→6)−*β*−D−glucopyranoside (**2**), the binding affinity value is −9.083 kcal/mol with three hydrogen bonds in the binding pocket of the 4FA6 protein at the amino acid residues Val 882 (2.52, 2.81 Å), Ser806 (2.98, 3.18 Å), and THR887 (2.68 Å). In particular, this compound interacts with amino acid residue Val882, showing high potential to inhibit the PI3K/mTOR protein. ILE963, ILE831, and MET953 are amino acid residues of the PI3K/mTOR protein that interacts hydrophobically with 5,7,4′−trihydroxyisoflavone 7−*O*−*β*−D−apiofuranosyl−(1→6)−*β*−D−glucopyranoside

### 2.2. Molecular Dynamic Simulation 

**Figure 3:**
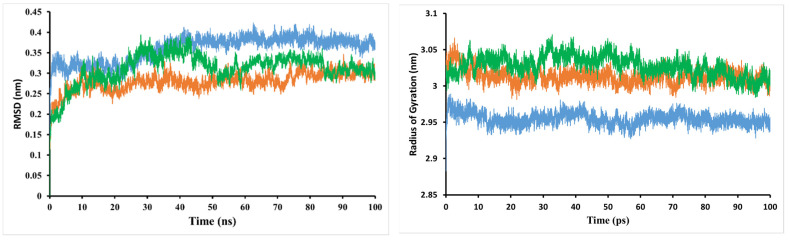
Root-mean-square deviation (RMSD) of the backbone atoms and radius of gyration (Rg) from the starting structure for *trans*−3−*O*−*p*−hydroxycinnamoyl ursolic acid (in blue), 5,7,4′−trihydroxyisoflavone 7−*O*−*β*−D−apiofuranosyl− (1→6)−*β*−*D*−glucopyranoside (in green), and the reference compound (in orange) with the PI3K/mTOR protein throughout 100 ns MD simulations.

MD simulation looks at the stability of inhibitor–target complexes, structural specifics, and orientational flexibilities, as well as the accuracy of inhibitor–target binding affinities [19]. To test the performance stability of the *trans*−3−*O*−*p*−hydroxycinnamoyl ursolic acid–PI3K/mTOR and PI3Kα/mTOR–PI3K/mTOR complexes, the root-mean-square deviation (RMSD) values of the backbone atoms of the entire system were evaluated (Figure 3). These complexes show variable bias during simulation in the backbone of PI3K/mTOR. After the equilibration period, the simulation system of the reference compound increased continuously from 0.2–0.3 nm; finally, after 20 ns, the system stabilized near 0.29 nm during the simulation. The RMSD value of *trans*−3−*O*−*p*−hydroxycinnamoyl ursolic acid in the early stages of the system was stable at 0.32 nm, continuously increasing from 0.32–0.38 nm at 20–40 ns; finally, after 40 ns, the system stabilized with an average of 0.38 nm over the whole simulation. According to the analysis above, the simulation validation of all systems has a small deviation from their initial configuration, and the RMSD values of the reference and the compound *trans*−3−*O*−*p*−hydroxycinnamoyl ursolic acid (**1**) are in the range of stability after MD simulation, such as 0.29 and 0.38 nm, respectively (Figure 3). 

The time evolution of weighted root-mean-square deviations (RMSDs) for backbone atoms of the PI3K/mTOR protein complex with compound 5,7,4′−trihydroxyisoflavone 7−*O*−*β*−D−apiofuranosyl−(1→6)−*β*−D−glucopyranoside (**2**) from their initial positions (t = 0) were calculated. As shown in Figure 3, the RMSD values of the complex increased during the simulation period of 0–40 ns and decreased during the period of 40–50 ns. The complex system is stable with RMSD values in the range of 0.3 nm to the end of the simulation.

The radius of gyration (Rg) was also used to evaluate the compactness of the protein–ligand complex [20,21]. When a ligand is bound, Rg can predict how the protein will unfold and fold. A high Rg value indicates that the protein–ligand association is not unfolded (less tight). The mean Rg value of the PI3K/mTOR complex with compound **1** was in the range of 2.88–2.99 nm, while between 2.98–3.07 nm for the reference compound. This implies that the PI3K/mTOR complex with the low Rg-valued compound **1** inhibitors shows less disorder/entropy than the PI3K/mTOR complex with the reference compound. The mean Rg value of the PI3K/mTOR complex with compound **2** was in the range of 2.98–3.06 nm.

### 2.3. SMD Results

**Figure 4:**
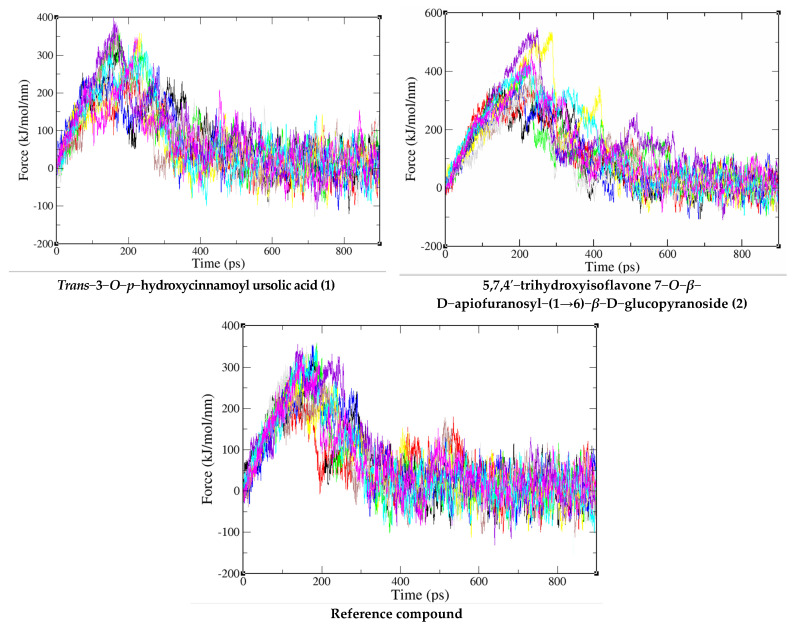
Force-time profiles of a representative SMD trajectory of compounds (**1** and **2**) and reference compound with PI3K/mTOR protein complex.

Through the SMD simulation, the profiles of pulling force over simulation time are shown in Figure 4 and Table 2. The pulling work spent on the co-crystallized ligand from the binding site of PI3K/mTOR was 72.0 ± 6.7 kcal/mol and F_max_ = 331.4 ± 30.4 pN. While the compound *trans*−3−*O*−*p*−hydroxycinnamoyl ursolic acid has W = 83.5 ± 10.6 kcal/mol and Fmax = 336.2 ± 45.3 pN, compound 5,7,4′−trihydroxyisoflavone 7−*O*−*β*−D−apiofuranosyl−(1→6)−*β*−D−glucopyranoside has 126.6 ± 21.7 kcal/mol and F_max_ = 430.3 ± 84.0 pN, which are higher than that of the reference compound. According to equation 2, we can estimate the non-equilibrium binding free energy ∆G_neq_^Jar^ = ΔG(t_end_) [22], which is equal to −66.89196, −69.86074 and −101.2317 kcal/mol for the reference compound—corresponding to PI3K/mTOR, *trans*−3−*O*−*p*−hydroxycinnamoyl ursolic acid, PI3K/mTOR and 5,7,4′−trihydroxyisoflavone 7−*O*−*β*−D−apiofuranosyl−(1→6)−*β*−D−glucopyranoside, and PI3K/mTOR complex, respectively. Since the pulling speed is substantially faster than that used in the experiment, ∆G_neq_^Jar^ has a very large value [23]. In the error bars, the F_max_ and W of the selected compounds are larger than the reference compound, indicating that *trans*−3−*O*−*p*−hydroxycinnamoyl ursolic acid and 5,7,4′−trihydroxyisoflavone 7−*O*−*β*−D−apiofuranosyl−(1→6)−*β*−D−glucopyranoside binds PI3K/mTOR more strongly than the reference compound. 

This conclusion is also supported by the results obtained for ∆G_neq_^Jar^, which are lower for 5,7,4′−trihydroxyisoflavone 7−*O*−*β*−D−apiofuranosyl−(1→6)−*β*−D−glucopyranoside and *trans*−3−*O*−*p*−hydroxycinnamoyl ursolic acid than for the reference compound. Furthermore, it is in agreement with the docking prediction, showing that the binding affinity for 5,7,4′−trihydroxyisoflavone 7−*O*−*β*−D−apiofuranosyl−(1→6)−*β*−D−glucopyranoside and *trans*-3-*O-p*-hydroxycinnamoyl ursolic acid are better than that of the reference compound (Figure 4). Therefore, the compounds *trans*−3−*O*−*p*−hydroxycinnamoyl ursolic acid and 5,7,4′−trihydroxyisoflavone 7−*O*−*β*−D−apiofuranosyl−(1→6)−*β*−D−glucopyranoside are predicted to be a very promising inhibitor against PI3K/mTOR and direct their cytotoxic activity against *Hepatocellular carcinoma*.

### 2.4. Biological Activities of Crude Extract, Fractions, and Isolated Compounds

#### 2.4.1. The *in Vitro* Cytotoxicity of Crude Extract, Fractions, and Isolated Compounds

The biological activity of crude extract (**MD**), fractions (ethyl acetate—**MDE** and water—**MDW**), and isolated compounds *trans*−3−*O*−*p*−hydroxycinnamoyl ursolic acid (**1**) and 5,7,4′−trihydroxyisoflavone 7−*O*−*β*−D−apiofuranosyl− (1→6)−*β*−D−glucopyranoside (**2**) from *Millettia dielsiana* were evaluated for *in vitro* cytotoxicity on the *Hepatocellular carcinoma* cell line (HepG2) as Table 3 shows.

The results indicated that the **MDW** fraction did not show cytotoxicity with IC_50_ values of over 100 µg/mL. The **MD** extract and **MDE** fraction demonstrated moderate cytotoxicity with IC_50_ values of 81.2 and 60.4 µg/mL, respectively. *Trans*-3−*O*−*p*−hydroxycinnamoyl ursolic acid (**1**) exhibited relative potentcy with an IC_50_ value of 23.1 μM. Especially, 5,7,4′−trihydroxyisoflavone 7−*O*−*β*−*D*−apiofuranosyl−(1→6)−*β*−D−glucopyranoside (**2**) demonstrated potent cytotoxicity against the HepG2 cell line with an IC_50_ value of 16.3 μM, which is lower than that of the positive control, paclitaxel (IC_50_ value of 45.1 μM). That showed the **MDE** fraction contained the most active ingredients and compounds (**1** and **2**) and could be the main component inhibiting against the HepG2 cell line. In addition, these initial results show a high correlation between in silico docking score and the experimental inhibition. It suggests that this computational model could be useful in the prediction of potential compounds with inhibition activity against the HepG2 cell line.

#### 2.4.2. The Antioxidant Activity on Liver Cells of Crude Extract, Fractions, and Isolated Compounds

The crude extract (**MD**), fractions (ethyl acetate, **MDE** and water, and **MDW**), and isolated compounds (**1** and **2**) from *Millettia dielsiana* were tested for in *vitro* antioxidant activity on mouse hepatocytes as Table 4 shows:

The **MDW** fraction displayed weak antioxidant activity with an ED_50_ value of 93.7 µg/mL. The **MD** extract and **MDE** fraction showed relatively potent antioxidant activity with ED_50_ values of 24.4 and 19.3 µg/mL, respectively. This is very meaningful in the process of drug research from medicinal herbs. The demonstration of the active fractions will guide further studies on their chemical compositions. The most potent was the ethyl acetate fractional extract, and compounds (**1** and **2**) may be the main components that have shown activity. Two isolated compounds, including *trans*-3-*O-p*-hydroxycinnamoyl ursolic acid (**1**) and 5,7,4′−trihydroxyisoflavone 7−*O*−*β*−*D*−apiofuranosyl-(1→6) −*β*−D−glucopyranoside (**2**), demonstrated potent antioxidant activity with ED_50_ values of 30.7 μM and 20.5 μM, respectively, which are higher than that of the positive control, curcumin (ED_50_ value of 7.2 μM). This adds further evidence of the hepatoprotective effects of *Millettia dielsiana.*

## 3. Materials and Methods

### 3.1. Plant Materials

The stems of plant samples were collected in Yen Son district, Tuyen Quang province, Vietnam in August 2019. They were identified by Dr. Nguyen Quoc Binh, Vietnam National Museum of Nature, VAST under the scientific name *Millettia dielsiana* Harms ex Diels. A voucher specimen was deposited at the Herbarium of the Institute of Chemistry, VAST.

### 3.2. Sample Processing, Extraction and Isolated Compounds (**1**–**2**) from Millettia dielsiana

The stems of *Millettia dielsiana* dried at 60 °C until they had a constant weight (5 kg). They were then cut into small pieces, powdered, and extracted with ethanol by sonication 3 times at room temperature. The combined extracts were then evaporated to give the total ethanolic residue (**MD**, 220 g), which was then suspended in water and successively partitioned with ethyl acetate to obtain an ethyl acetate fraction (**MDE**, 60 g) and an ethanol fraction (**MDW**, 150 g) after removal of solvent in a vacuum.

The ethyl acetate fraction (**MDE**, 50 g) was chromatographed on a silica gel column and eluted with dichloromethane/methanol (50/1→ 1/1) to obtain five fractions (**MDE1**→ **MDE5**). The **MDE4** (3.2 g) fraction was chromatographed on an RP-18 column and eluted with methanol/water (3:7→ 7:3) to obtain three fractions (**MDE4.1**→ **MDE4.3**). The **MDE4.3** fraction was subjected on a Sephadex LH-20 and eluted with methanol to yield compound **1** (6.3 mg). **MDE5** fraction was chromatographed on an RP-18 column and eluted with methanol/water (3:7→ 7:3) to obtain four fractions **(MDE5.1**→ **MDE5.4**). The **MDE5.2** fraction was chromatographed on a silica gel column and eluted with dichloromethane/methanol (7/1→ 1/1) to yield compound **2** (5.5 mg).

*Trans*−3−*O*−*p*−hydroxycinnamoyl ursolic acid (**1**): Colorless powder. ^1^H-NMR (500 MHz, CDCl_3_) δ: 7.58 (1H, d, *J* = 15.5 Hz, H-3’), 7.39 (2H, d, *J* = 8.0 Hz, H-2”, 6”), 6.80 (2H, d, *J* = 8.0 Hz, H-3”, 5”), 6.30 (1H, d, *J* = 15.5 Hz, H-2’), 5.42 (1H, m, H-12), 4.49 (1H, m, H-3α), 2.17 (1H, m, H-18), 1.23 (3H, s, H-23), 1.09 (3H, s, H-27), 0.99 (3H, s, H-26), 0.92 (3H, s, H-24), 0.91 (3H, d, *J* = 5.0 Hz, H-29), 0.86 (3H, d, *J* = 7.0 Hz, H-30), and 0.78 (3H, s, H-25). ^13^C-NMR (125 MHz, CDCl_3_) δ: 39.2 (C-1), 27.1 (C-2), 81.1 (C-3), 39.6 (C-4), 55.9 (C-5), 18.8 (C-6), 33.6 (C-7), 40.1 (C-8), 50.0 (C-9), 37.5 (C-10), 22.2 (C-11), 123.6 (C-12), 138.4 (C-13), 42.5 (C-14), 28.6 (C-15), 25.0 (C-16), 46.1 (C-17), 53.4 (C-18), 39.5 (C-19), 39.4 (C-20), 31.2 (C-21), 37.3 (C-22), 28.1 (C-23), 16.1 (C-24), 16.0 (C-25), 17.6 (C-26), 23.9 (C-27), 182.3 (C-28), 17.5 (C-29), 21.6 (C-30), 168.5 (C-1’), 118.3 (C-2’), 143.6 (C-3’), 129.2 (C-1”), 130.4 (C-2”, 6”), 117.1 (C-3”, 5”), and 159.0 (C-4”). The above data were identical to the literature data [24].

**Figure 5:**
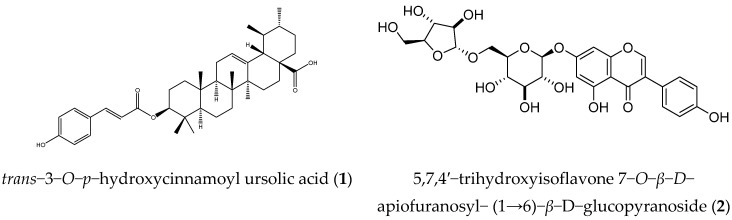
Structures of compounds **1** and **2**.

*5,7,4*’−*trihydroxyisoflavone 7*−*O*−*β*−*D*−*apiofuranosyl*−*(1→6)*−*β*−*D*−*glucopyranoside* (**2**): Colorless powder. ^1^H-NMR (500 MHz, CDCl_3_) δ: 8.13 (1H, s, H-2), 7.39 (2H, d, *J* = 8.5 Hz, H-2”, 6”), 6.85 (2H, d, *J* = 8.5 Hz, H-3”, 5”), 6.71 (1H, d, *J* = 2.0 Hz, H-8), 6.51 (1H, d, *J* = 2.0 Hz, H-6). *Glucose*: 4.97 (1H, d, *J* = 5.0 Hz, H-1), 3.49 (1H, m, H-2), 3.51 (1H, m, H-3), 3.32 (1H, m, H-4), 3.65 (1H, m, H-5), 3.61 (1H, dd, *J* = 12.0 and 5.0 Hz, H-6α), 4.05 (1H, dd, *J* = 12.0 and 1.0 Hz, H-6*β*). *Apiose:* 4.90 (1H, d, *J* = 2.5 Hz, H-1), 3.93 (1H, d, *J* = 2.5 Hz, H-2), 3.75 (1H, d, *J* = 12.0 Hz, H-4α), 4.04 (1H, d, *J* = 12.0 Hz, H-4*β*), and 3.52 (1H, s, H-5). ^13^C-NMR (125 MHz, CD_3_OD) δ: 155.4 (C-2), 125.0 (C-3), 182.5 (C-4), 163.5 (C-5), 101.2 (C-6), 164.7 (C-7), 96.1 (C-8), 159.2 (C-9), 108.1 (C-10), 123.2 (C-1’), 131.4 (C-2’), 116.3 (C-3’), 158.9 (C-4’), 116.3 (C-5’), 131.4 (C-6’). *Glucose*: 101.7 (C-1), 74.7 (C-2), 77.9 (C-3), 71.7 (C-4), 77.2 (C-5), 69.1 (C-6). *Apiose*: 111.2 (C-1), 78.2 (C-2), 80.5 (C-3), 75.1 (C-4), and 65.8 (C-5). The above data were identical to the literature data [25]. The structures of compounds **1** and **2** are shown in Figure 5.

### 3.3. Isolated and Determined Chemical Structures Methods

Compounds were isolated and purified by using a combination of various chromatographic methods involving thin-layer chromatography (TLC), preparative thin-layer chromatography (PTLC), column chromatography (CC) on different stationary phase such as silica gel, YMC RP-18, and Sephadex LH-20.

The chemical structures of isolated compounds were elucidated by a combination of physical parameters and modern spectroscopic methods, such as melting point (mp), nuclear magnetic resonance spectroscopy methods including 1D-NMR (^1^H-NMR, ^13^CNMR, and DEPT) and 2D-NMR (HSQC and HMBC), and combined comparison with references.

### 3.4. Biological Activity Test Methods

#### 3.4.1. Cytotoxicity Test Method

The in vitro cytotoxicities of the compounds (**1** and **2**) against hepatic cancer (HepG2) were tested with an MTT [3−(4,5−dimethylthiazol−2−yl)−2,5−diphenyltetrazolium bromide] assay in the Institute of Natural Products Chemistry, Vietnam. The MTT-based colorimetric method was applied to evaluate the effect of compounds on the survival of HepG2 cancer cells. Cell lysates were dripped onto 96-well microplates (1.5 × 10^5^ cells/well) and then incubated with test compounds in a concentration range of 50→ 1 µg/mL (µM), each concentration repeated 3 times. Ellipticine or Paclitaxel in DMSO served as positive controls (+). The optical density was measured at λ = 540/720 nm on an Infinite F50 instrument (Tecan, Männedorf, Switzerland). The results were read on an ELISA machine at 495–515 nm [26].

#### 3.4.2. The Antioxidant Activity on Liver Cells Test Method

The antioxidant activity of the test compounds was demonstrated by reducing the amount of H_2_O_2_, resulting in a decrease in the color of the reaction between H_2_O_2_ and phenol red on liver cells. Hepatocytes from mouse liver of BALB/c after isolation were stabilized for 1–2 days and then put in a 96-well plate with density of 1 × 10^4^ cells/well to grow overnight in a 5% CO_2_ incubator at 37 °C. Test compounds or curcumin (positive control) were added at different concentrations. Then, the optical density (OD value—Optical Density) of formazan formed was measured with Microplate Reader at 492 nm. All experiments were repeated 3 times. The free radical scavenging effect was assessed with the ED_50_ value [27].

### 3.5. Preparation of Target Protein and Compounds

The crystal structure of PI3K/mTOR was downloaded from the Protein Data Bank (PDB ID: 4FA6) [28] with a resolution of 2.70 Å. After downloading, unnecessary parts of the target protein for molecular docking simulation such as the model of water-crystallized solvent, dissolved ions, and co-crystallized ligands were removed with Discovery Studio 2021 [29]. A set of 50 compounds isolated from *Millettia dielsiana* was collected from the previous literature as Table S1 presents [11,12,13]. The geometric structures of the compounds were built with MarvinSketch software [30] and optimized with an MMFF94s force field [31].

### 3.6. Molecular Docking

Molecular docking simulation to introduce research compounds into the active site of PI3Kα/mTOR was performed with the Autodock Vina v1.2.3 program [32]. The *pdbqt files containing coordinate information, partial charge, and atom type were prepared using the AutodockTools 1.5.6 suite. The ligand is regarded as flexible, while the protein is considered rigid. A grid point 21 × 21 × 21 with a gap of 1 was set up, and the grid center coordinates x = 44.5 Å, y = 15.1 Å, z = 31.3 Å were taken according to the center of the co-crystallized ligand. The exhaustiveness value was set to a high-performance level of 400 to increase the accuracy of the molecular docking simulation. To evaluate the docking simulation approach, re-docking was also performed. The co-crystallized ligand was docked back to the binding pocket in the protein and its site for comparison with RMSD [33]. Each protein–ligand complex is tested for hydrophobic and hydrogen bond interactions. Two– and three–dimensional interaction images were obtained using Discovery Studio 2021 as visualizing software [29].

### 3.7. Molecular Dynamic Simulation

GROMACS 2020.1 [34] was applied to conduct molecular dynamics (MD) simulations for the compound with the best binding affinity after molecular docking simulation and to control PI3Kalpha/mTOR-IN-1 in complex with PI3Kα/mTOR for a period of 100 ns. Molecular dynamics (MD) simulation was used to evaluate the stability and study the dynamic behavior of the PI3Kα/mTOR protein with the research compound. The topology of the PI3Kα/mTOR receptor structure was constructed using the pdb2gmx tool in combination with the AMBER-f99SB-ILDN force field and TIP3P water model. While the topology files of the inhibitor molecules were generated based on the general Amber force field with AmberTools 19 [35] and converted the AMBER force field format to GROMACS format using the Acpype [36] protocol. A restrained electrostatic potential charge (RESP) model was used to specify the atomic partial charges of the inhibitors investigated at B3LYP/6-31G* using GAMESS [37,38]. Docked PI3Kα/mTOR complexes were placed in the center of the triclinic box with center coordinates x = 5954, y = 4655, z = 5222 (nm) and box size 11,909 nm × 9310 nm × 10,443 nm. After that, the studied system was neutralized through suitable counterions when the total charge of the PI3Kα/mTOR–inhibitor complex was -8 e. The time steps were set at 2 fs. To calculate the long-range electrostatic interactions of all systems, the particle mesh Ewald (PME) method was used with a cutoff point of 0.9 nm [39]. 

The van der Waals forces are calculated with the same cutoff point. Energy minimization for the MD simulation system was performed using the steepest descent algorithm. The integrator was controlled with V–rescale and Parrinello–Rahman algorithms to maintain an absolute temperature of 300K and a pressure of 1 bar of the system. In the MD simulations, the C–alpha atoms were position constrained. Simulation of NVT and NPT sets at 0.5 ns and 2.0 ns respectively were calculated to model equilibrium systems. Finally, 100 ns MD simulations were performed with no position restriction at 300 K and 1 bar. Analysis of simulated trajectories were obtained by calculating values of RMSD and RMSF, and then visualized using VMD v1.9.4 [40].

### 3.8. Steered Molecular Dynamic (SMD)

The final simulation structure was prepared as the input file for the SMD simulations. Under the action of external forces, the receptor can drift, so to prevent this, C-α was restricted to using a harmonic potential with a spring constant of 1000 kJ/nm/mol. In the SMD simulation, an external regulatory force F acting on the center of mass of the ligand pulled the ligand away from the binding site of PI3Kα/mTOR along the *Z*–axis direction determined by the MSH (minimal steric hindrance) method.. This force expression is calculated F = k_s_(vt–z), where z is the displacement of the spring-bound ligand center of mass in the direction pulling away from the initial position, the pulling speed v = 5 nm/ns, and, as in the AFM experiment [41], the spring constant of the cantilever was chosen to be k_s_ = 600 kJ/mol/nm^2^. The force–time profiles obtained in the SMD simulation were used to calculate the non-equilibrium work according to the following equation:

(1) $$W=v\overset{t}{\underset{0}{\int }}F\left(t\right)dt$$

We performed 10 SMD trajectories for each protein–ligand complex and recorded the external pull and ligand shift every 0.1 ps of each orbital of the PI3Kα/mTOR–inhibitor complex for better sampling. The Jarzynski equation [42,43] was used to estimate the non-equilibrium binding free energies from the simulated SMD trajectories.

(2) $$exp\left(\frac{-\Delta G}{k_{B}T}\right)=\langle exp(-\frac{W_{t}-\frac{1}{2}k{\left(z_{t}-vt\right)}^{2}}{k_{B}T})\rangle$$

## 4. Conclusions

The results of the study have initially elucidated the hepatoprotective activity of extracts and isolated compounds from *Millettia dielsiana*. Complementary experimental evidence is needed to bring support to a renewed interest for *Millettia dielsiana* as a potential hepatoprotective activity agent candidate.

Fifty compounds isolated from *Millettia dielsiana* were docked into the binding pocket of the PI3K/mTOR protein. Compounds (**1** and **2**) were considered the best-docked compound with low binding affinity (−9.237 and −9.083 kcal/mol), hydrogen bond formation with important amino acids, and hydrophobic interactions with various amino acid residues, showing high potential to inhibit the PI3K/mTOR protein. In molecular dynamic simulation, the RMSD value of the PI3K/mTOR complex system with compound **1** stabilized with an average of 0.38 nm over the whole simulation after 40 ns, and the complex system of compound **2** was stable with RMSD values near 0.3 nm to the end of the simulation. The radii of gyration value of the PI3K/mTOR complex with compounds **1** and **2** were in the range of 2.88–2.99 nm and 2.98–3.06 nm, respectively, while the measure was between 2.98–3.07 nm for the reference compound. In the SMD simulation, compounds **1** and **2** were predicted to be very promising inhibitors against PI3K/mTOR and direct their cytotoxic activity against *Hepatocellular carcinoma*.

In vitro, the crude extract (**MD**) and the ethyl acetate (**MDE)** fraction demonstrated moderate cytotoxicity with IC_50_ values of 81.2 and 60.4 µg/mL, respectively, and compounds (**1** and **2**) exhibited potency on the *Hepatocellular carcinoma* cell line (HepG2) with IC_50_ values of 23.1 μM and 16.3 μM, respectively. The **MD** extract and **MDE** fraction showed relatively potent antioxidant activity with ED_50_ values of 24.4 and 19.3 µg/mL, respectively, and compounds (**1**–**2**) demonstrated potent antioxidant activity with ED_50_ values of 30.7 μM and 20.5 μM, respectively.

## Figures and Tables

**Table 1:** The achieved docking results for the complexes of the studied compound and reference compound (PI3Kalpha/mTOR-IN-1) with PI3Kα/mTOR the target protein.

Compounds	Binding Affinity (kcal/mol)	Hydrogen Bond		Hydrophobic Interactions
		With Amino Acid Residues	Bond Length (Å)	
**1**	−9.237	Val 882	2.45	Ala 805, Ile 963, Lys833, Lys808, Ile 831, Pro 810, Met 804, Met 953
**2**	−9.083	Val 882	2.52, 2.81	ILE963, ILE831, MET953
		Ser806	2.98, 3.18	
		THR887	2.68	
**Ref.**	−9.12	Val 882	1.92, 2.16	Phe 961, Tyr 867, Ile 963, Ile 879, Ile 831, Met 804

**Table 2:** SMD Results from 20 independent trajectories.

No.	Compounds	F_max_ (pN)	W (kcal/mol)	∆G_neq_^Jar^ (kcal/mol)
1	**1**	336.2 ± 45.3	83.5 ± 10.6	−69.86074
2	**2**	430.3 ± 84.0	126.6 ± 21.7	−101.2317
3	PI3Kalpha/mTOR-IN-1	331.4 ± 30.4	72.0 ± 6.7	−66.89196

**Table 3:** Test results of *in vitro* cytotoxicity of crude extract, fractions, and compounds (**1** and **2**).

No.	Samples	HepG2 Cell Lines	
		Cell Growth Inhibition Rate (%) *	IC_50_ Values
1	**MD**	55.3 ± 0.4	81.2 µg/mL
2	**MDE**	59.8 ± 0.2	60.4 µg/mL
3	**MDW**	30.5 ± 0.9	>100 µg/mL
4	**1**	66.4 ± 1.0	23.1 μM
5	**2**	73.2 ± 0.6	16.3 μM
6	Paclitaxel 50 μM	54.2 ± 1.8	45.1 μM

* The maximum test concentration should be 100µg/mL for crude extract and fractions, and 50 µg/mL for isolated compounds. Values are means (*n* = 9) ± SD; *p* < 0.05.

**Table 4:** Test results of in vitro antioxidant activity of crude extract, fractions, and compounds (**1** and **2**).

No.	Samples	Liver Cell Survival Rate (%) *	ED_50_ Values
1	**MD**	60.9 ± 1.1	24.4 µg/mL
2	**MDE**	70.2 ± 0.7	19.3 µg/mL
3	**MDW**	26.6 ± 1.8	93.5 µg/mL
4	**1**	58.5 ± 0.9	30.7 μM
5	**2**	66.2 ± 1.4	20.5 μM
6	Curcumin 50 μM	73.8 ± 1.2	7.2 μM

* The maximum test concentration should be 100µg/mL for crude extract, fractions and 50 µg/mL for isolated compounds. Values are means (*n* = 9) ± SD; *p* < 0.05.

## Data Availability

Not applicable.

## Supplementary Materials

The following supporting information can be downloaded at https://www.mdpi.com/article/10.3390/molecules27248978/s1, Table S1: In silico Docking of 50 isolated compounds from *Millettia dielsiana* against PI3K/mTOR.
