# Supplementary material for: Hepatoprotective Effect of Millettia dielsiana: In Vitro and In Silico Study
Source: Molecules. 2022 Dec 16;27(24):8978. doi: 10.3390/molecules27248978 (PMC9787921; doi:10.3390/molecules27248978)
Supplement: Supplementary file 1 [file molecules-27-08978-s001.zip › molecules-2023630-supplementary.pdf]

molecules

Supplementary Materials

Hepatoprotective Effect of *Millettia dielsiana*: In Vitro and In Silico Study

Vu Thi Thu Le [1], Dao Viet Hung [1], Bui Minh Quy [2], Pham Thi Hong Minh [3,4] and Do Tien Lam [3,4,*]

[1] Thai Nguyen University of Agriculture and Forestry, Thai Nguyen University (TNU), Quyet Thang, Thai Nguyen 24119, Vietnam
[2] Thai Nguyen University of Sciences, Thai Nguyen University (TNU), Tan Thinh, Thai Nguyen 24119, Vietnam;
[3] Institute of Natural Products Chemistry, Vietnam Academy of Science and Technology (VAST), 18 Hoang Quoc Viet, Cau Giay, Hanoi 10072, Vietnam
[4] Graduate University of Science and Technology, Vietnam Academy of Science and Technology (VAST), 18 Hoang Quoc Viet, Cau Giay, Hanoi 10072, Vietnam
* Correspondence: dotienlam198@gmail.com; Tel.: +84-9-8985-6515

Table S1: *In silico* Docking of 50 isolated compounds from *Millettia dielsiana against* PI3K/mTOR

No.	Symbol	Name of compounds	ΔG (kcal/mol)	Structural Formula
1	D15 (**1**)	*Trans*-3-*O*-*p*-hydroxycinnamoyl ursolic acid	-9.237	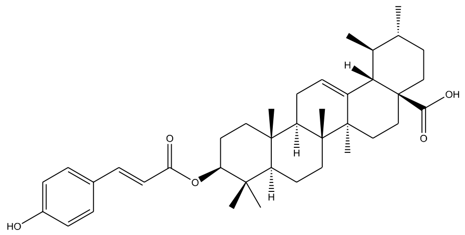
2	D50 (**2**)	5,7,4'-trihydroxyisoflavone 7-*O*-*β*-D-apiofuranosyl-(1→6)-*β*-D-glucopyranoside	-9.083	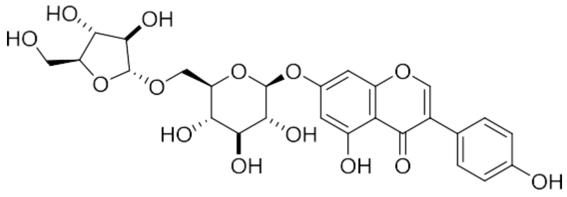
3	D49	Genistin	-8.914	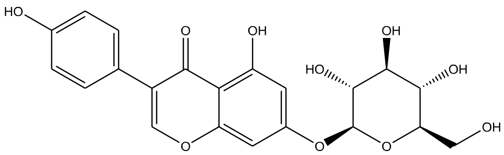

4	D40	Daidzin	-8.754	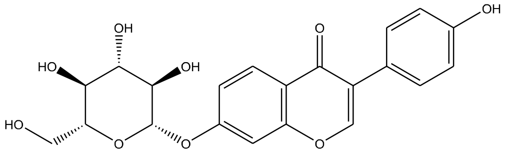
5	D20	Durmillone	-8.445	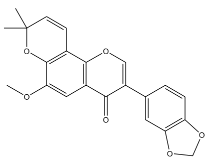
6	D38	Millesianin F	-8.372	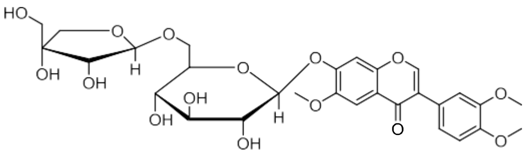
7	D42	7-hydroxy-4’,6 dimethoxyisoflavone-7-*O*-*β*-D-apiofuranosyl-(1→6)-*β*-D-glucopyranoside	-8.307	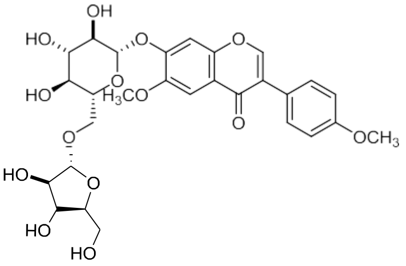
8	D31	Millesianin D	-8.213	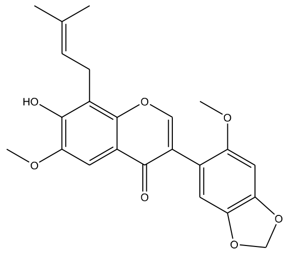
9	D39	Millesianin G	-8.209	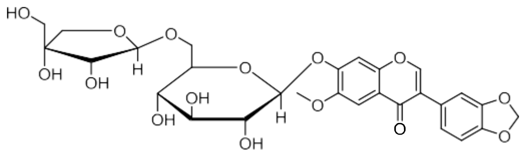
10	D23	Hydroxy-6-methoxy-3-4-methylenedioxy-8-3-3-dimethylallyl-isoflavone	-8.138	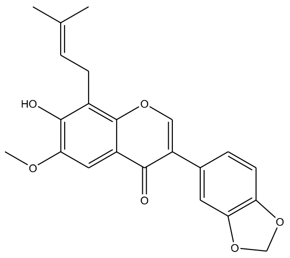

11	D45	7-hydroxy-4',8-dimethoxyisoflavone 7-*O*-*β*-D-apiofuranosyl-(1→6)-*β*-D-glucopyranoside	-8.127	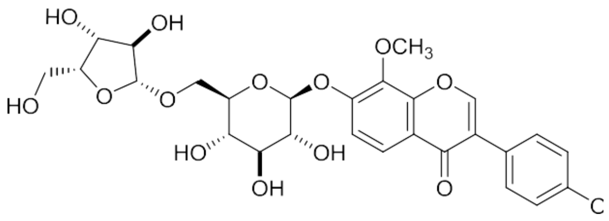
12	D28	Millesianin A	-8.074	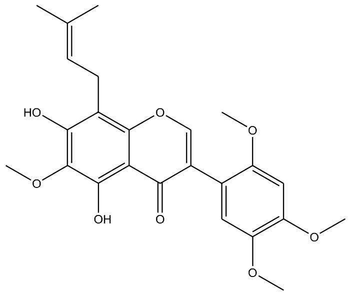
13	D19	Ichthynone	-8.067	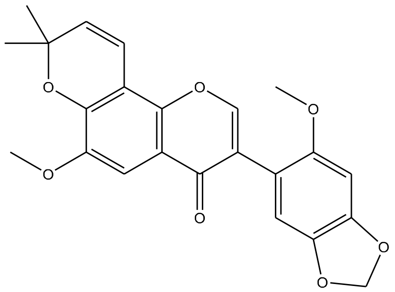
14	D25	Millesianin D	-8.046	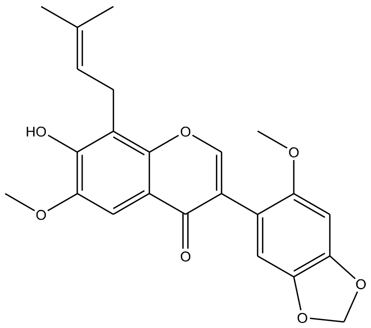
15	D47	Odoratin-7-*O*-*β*-D-glucopyranoside	-7.981	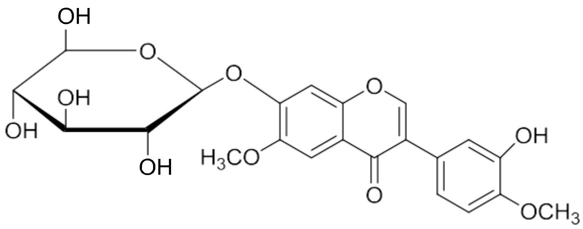

16	D22	Calopogonium isoflavone A	-7.969	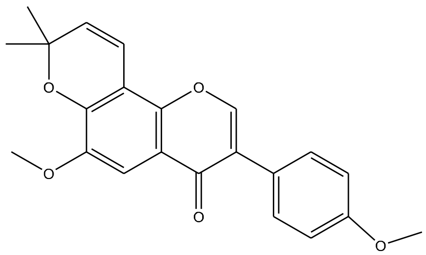
17	D32	Millesianin E	-7.968	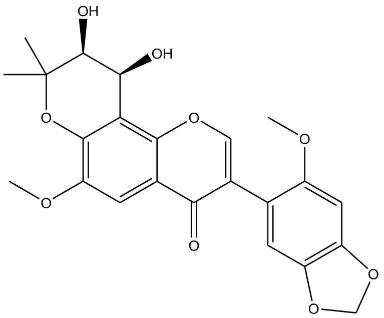
18	D44	7-hydroxy-4’,8-dimethoxyisoflavone-7-*O*-*β*-D-glucopyranoside	-7.929	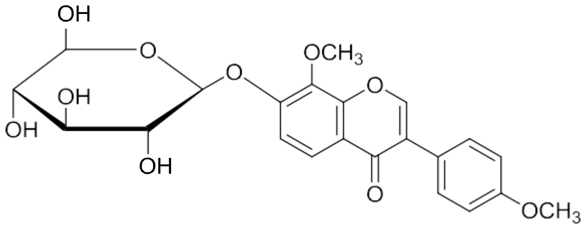
19	D41	Claclrastin-7-*O*-*β*-D-glucopyranoside	-7.927	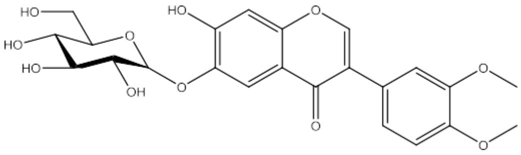
20	D5	Liquiritigenin	-7.825	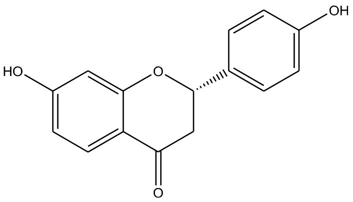
21	D4	Isoliquiritigenin	-7.822	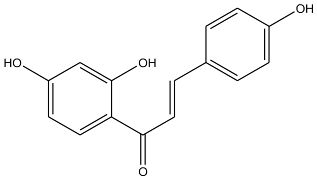

22	D43	Wistin	-7.803	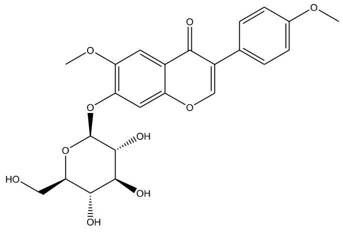
23	D18	Barbigerone	-7.721	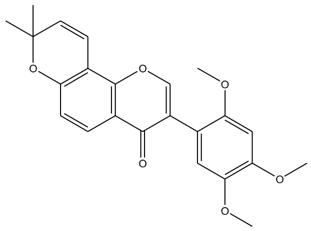
24	D10	Tupichinol C	-7.67	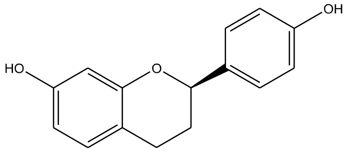
25	D46	Glycitin	-7.652	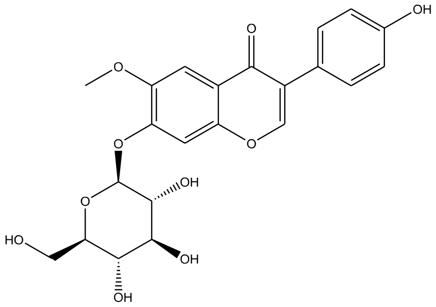
26	D30	Millesianin C	-7.631	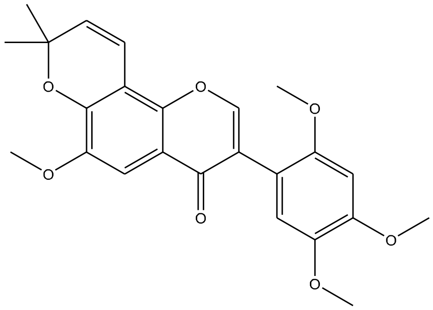
27	D33	Afromosin	-7.627	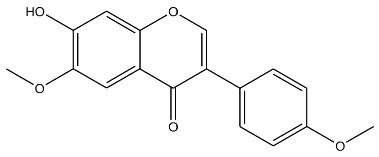

28	D35	Cladrastin	-7.623	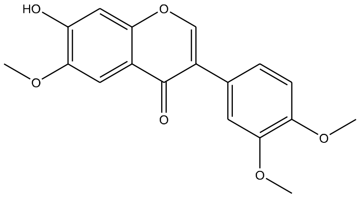
29	D16	Millesianin C	-7.618	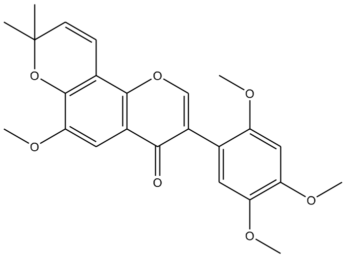
30	D48	Dalpatin	-7.582	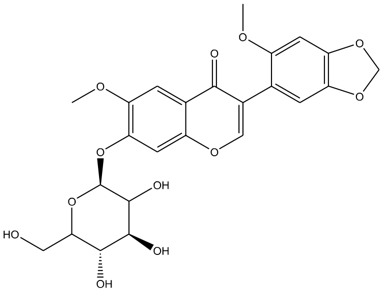
31	D21	Methoxycalpogonium isoflavone A	-7.557	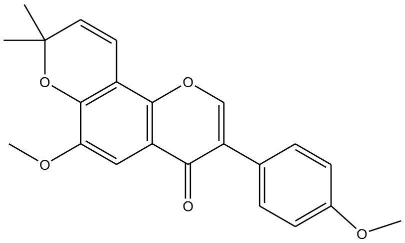
32	D2	Formononetin	-7.512	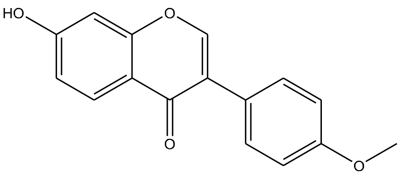
33	D17	Durallone	-7.482	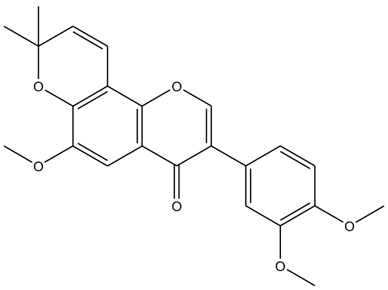

34	D6	Naringenin	-7.478	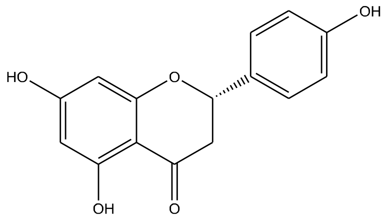
35	D27	Dielsianone	-7.475	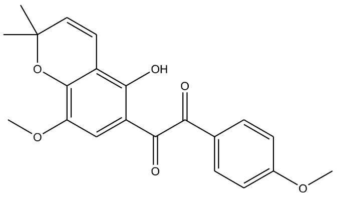
36	D29	Millesianin B	-7.347	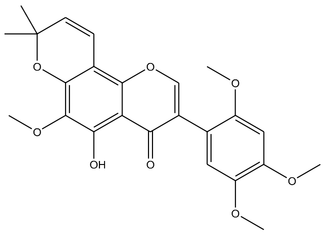
37	D8	Catechin	-7.316	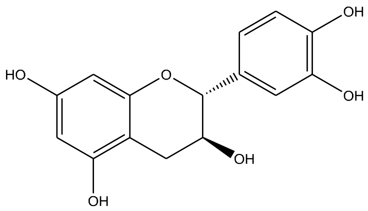
38	D11	(+)-Epicatechin	-7.241	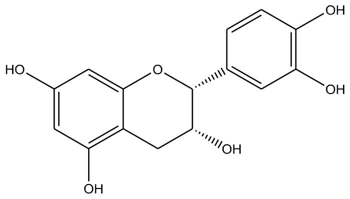
39	D36	Daidzein	-7.214	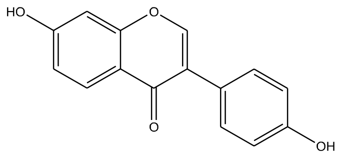
40	D3	Ononin	-7.192	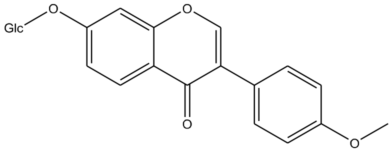

41	D7	Gallocatechin	-7.069	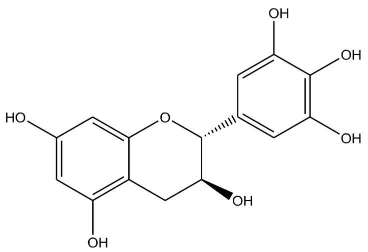
42	D37	Hernancorizin	-6.995	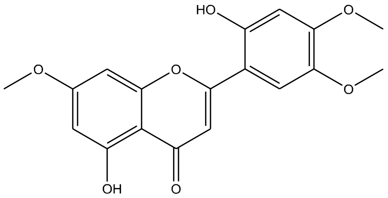
43	D24	Millesianin H	-6.826	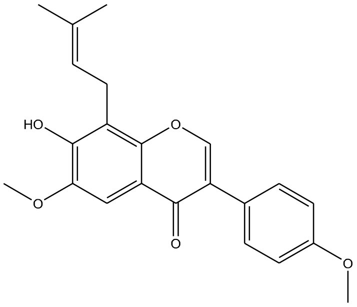
44	D1	Mildiside A	-6.808	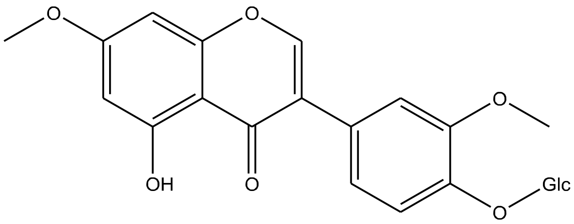
45	D34	Caviunin	-6.741	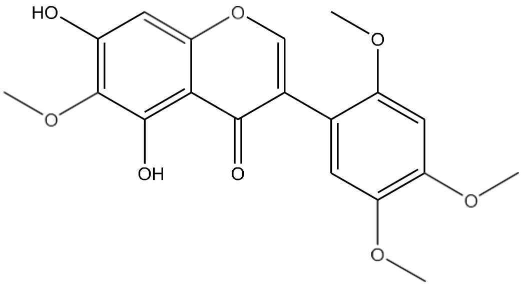
46	D12	(-)-Epicatechin	-6.736	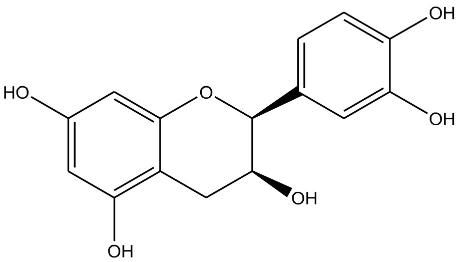

47	D26	Millesianin I	-6.718	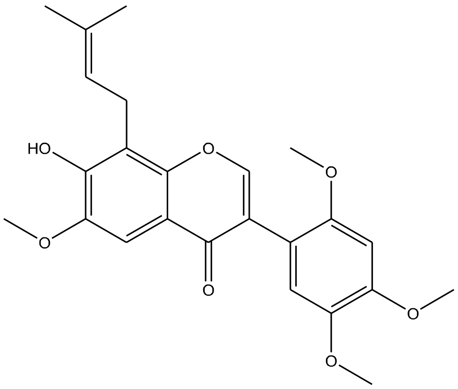
48	D9	(3*S*)-Vestitol	-6.467	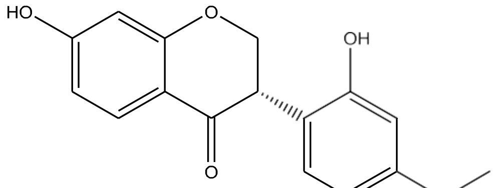
49	D14	*Trans*-Ferulic acid	-6.193	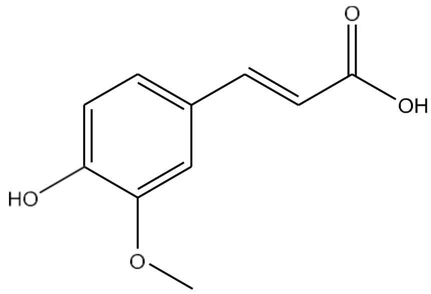
50	D13	Protocatechuic acid	-5.046	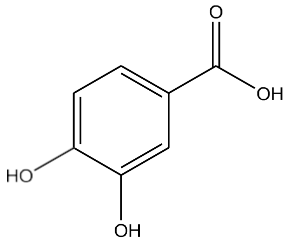
51	Ref.	PI3Kalpha/mTOR-IN-1	-9.12	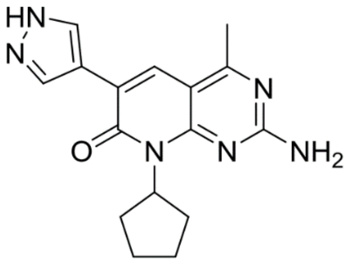
